# Latency-Based and Psychophysiological Measures of Sexual Interest Show Convergent and Concurrent Validity

**DOI:** 10.1007/s10508-017-1133-z

**Published:** 2017-12-20

**Authors:** Caoilte Ó Ciardha, Janice Attard-Johnson, Markus Bindemann

**Affiliations:** 1 School of Psychology, Keynes College, University of Kent, Canterbury, CT2 7NP UK; 2 Department of Psychology, Bournemouth University, Poole, UK

**Keywords:** Sexual interest, Viewing time, Reaction time, Pupil dilation, Pedophilia

## Abstract

Latency-based measures of sexual interest require additional evidence of validity, as do newer pupil dilation approaches. A total of 102 community men completed six latency-based measures of sexual interest. Pupillary responses were recorded during three of these tasks and in an additional task where no participant response was required. For adult stimuli, there was a high degree of intercorrelation between measures, suggesting that tasks may be measuring the same underlying construct (convergent validity). In addition to being correlated with one another, measures also predicted participants’ self-reported sexual interest, demonstrating concurrent validity (i.e., the ability of a task to predict a more validated, simultaneously recorded, measure). Latency-based and pupillometric approaches also showed preliminary evidence of concurrent validity in predicting both self-reported interest in child molestation and viewing pornographic material containing children. Taken together, the study findings build on the evidence base for the validity of latency-based and pupillometric measures of sexual interest.

## Introduction

For many research topics relating to sexuality and sexual behavior, it is sufficient for participants to self-report their sexual orientation or sexual interests. A large number of self-report tools are available to researchers for this purpose (Savin-Williams & Vrangalova, 2013). However, for research examining factors such as the cognitive mechanisms of sexual interests (e.g., Ortigue & Bianchi-Demicheli, 2008), it becomes important to be able to measure those interests less directly, or even implicitly. Additionally, there are applied contexts where understanding individuals’ sexual interests is clinically important but potentially vulnerable to social desirability biases—for example in the case of individuals who may have committed sexual offenses (see Kalmus & Beech, 2005). For this reason, much of the basic research that has examined the validity of indirect measures of sexual interest has done so with a distal or proximal goal of improving measurement of sexual interest relevant to forensic contexts, especially relating to sexual offending against children. In the current study, we examined the validity of multiple methods of assessing sexual interest among male participants, again with the distal goal of improving forensic assessments, but with relevance for broader sexology research.

Despite perceptions to the contrary, not all individuals who commit child sexual abuse have a sexual interest in children (Seto, 2008). While it may not be a universal treatment need, sexual interest in children is a leading predictor of sexual reoffending among those convicted of a sexual offense (Hanson & Morton-Bourgon, 2004). Accurately assessing sexual interest is, therefore, vital for targeting criminogenic treatment needs within rehabilitation and for estimating and managing risk. The current ‘gold standard’ in assessing sexual interest is by measuring genital responses, typically using penile plethysmography (PPG). A recent meta-analysis by McPhail et al. (2017) found that PPG is a valid measure of pedohebephilic interest, but that validity varies depending on PPG methodology. Historically, PPG has faced questions of validity, reliability, and admissibility in court (for a review, see Laws, 2009). PPG is not routinely used, for example, by Her Majesty’s Prison and Probation Service in the UK in the assessment of individuals who have committed sexual offenses. Where genital arousal is used in sex research more broadly, there is a risk to the representativeness and generalizability of findings due to volunteer bias (Morokoff, 1986; Wolchik, Braver, & Jensen, 1985). Practitioners and researchers carrying out assessments of sexual interest, therefore, need other indicators of sexual interest to complement or provide an alternative to the use of PPG.

As a response to the need for additional valid measures of sexual interest, researchers have tested response latency-based tasks (see Schmidt, Banse, & Imhoff, 2015; Thornton & Laws, 2009). The most established task uses a viewing time paradigm (Abel, Huffman, Warberg, & Holland, 1998) and is commercially available as a technique for measuring sexual interest (for a meta-analysis of viewing time research, see Schmidt, Babchishin, & Lehmann, 2017). Viewing time is based on the observation that individuals will spend longer looking at images they find sexually appealing than control or unappealing images. The task typically asks participants to rate images for attractiveness while simultaneously measuring response latency. Slowing down of response times is interpreted as indicative of an individual’s sexual interests. While the task is typically understood in relation to Singer’s (1984) theory of an *aesthetic* first phase in sexual arousal, the mechanisms underpinning this relatively robust phenomenon are currently poorly understood (Imhoff et al., 2010). There are concerns about the degree to which viewing time tasks may be open to manipulation. Schmidt et al. (2017) expressed surprise that there is a lack of research on the fakeability of viewing time. Additionally, for those undertaking assessment in adversarial settings, detailed task descriptions are available online; therefore, viewing time alone is not likely to provide a truly reliable measure of sexual interest for forensic assessment.

Other latency-based measures of sexual interest typically demand that the participant carries out a task quickly and accurately, while being presented with stimuli that may be related to their sexual interests. Due to the fact that a rapid response is required in these tasks, we use the term *reaction time tasks* to refer to them collectively and to differentiate them from viewing time (Maletzky, 2003). In these tasks, sexually salient stimuli may hinder or facilitate task performance in a way that is indicative of participants’ sexual interests. The Implicit Association Test (IAT) applied to the measurement of sexual interest asks participants to categorize stimuli as quickly as they can use two buttons (for a meta-analysis, see Babchishin, Nunes, & Hermann, 2013). In the trials of interest, each button has a concept category and an attribute category assigned to it. If the participant holds an association between the paired categories, they should experience a facilitation effect when required to categorize stimuli. For example, Nunes, Firestone, and Baldwin (2007) found that, when compared with other inmates, individuals who had committed sexual offenses against children were quicker to categorize stimuli when paired categories were child/sexy and adult/not sexy than when the pairs were in the opposite arrangement.

While the IAT hinges on a facilitation effect, other reaction time tasks depend on stimuli interfering with the primary task demands, thus suggesting that they hold some salience for the participant. A variant of the choice reaction time (CRT) task has been used where participants are instructed to report the location of a dot on a screen while ignoring potentially sexually appetitive stimuli on which that dot is overlaid. The CRT task typically results in longer reaction times to stimuli consistent with participants’ sexual interests (e.g., Mokros, Dombert, Osterheider, Zappalà, & Santtila, 2010; Wright & Adams, 1994). Similarly, the modified Stroop task asks participants to name the color of stimuli while ignoring their content. Pictorial (Ó Ciardha & Gormley, 2012) and word (Price & Hanson, 2007; Smith & Waterman, 2004) versions of the modified Stroop task have shown an interference effect of stimulus content when that content is sexually salient. The current study focuses on viewing time, IAT, CRT, and pictorial modified Stroop (P-MST) tasks as latency-based measures of sexual interest (for a broader review, see Schmidt et al., 2015). Compared to men, women—especially heterosexual women—do not demonstrate the same level of category-specific responding in physiological or latency-based tasks measuring sexual interest (Snowden & Gray, 2013). For this reason, combined with the lower prevalence rate of female-perpetrated sexual abuse, we focus only on the validity of tasks measuring sexual interest among men.

No single latency-based task currently holds a sufficient weight of evidence regarding its validity and reliability to be fully acceptable as a clinically useful measure of sexual interest. Indeed, case formulation is likely to be more reliably informed by a confluence of clinically relevant information from multiple assessment methods rather than by identifying the single best performing task (Schmidt et al., 2017). Therefore, the question of convergent and predictive/concurrent validity is of primary importance in building an evidence base for the clinical use of measures of sexual interest.

Several studies have, to date, examined the convergent validity of different latency-based measures relating to sexual interest. Meta-analysis (Schmidt et al., 2017) based on a handful of studies has shown associations with small to medium effect sizes between viewing time and self-report, IAT, PPG, and the Screening Scale for Pedophilic Interests (Seto & Lalumière, 2001). An earlier meta-analysis examining IAT research (Babchishin et al., 2013) reported relationships with moderate effect sizes with viewing time, self-report, and the Screening Scale for Pedophilic Interests.

Rönspies et al. (2015) compared viewing time with CRT, and an adaptation of the Implicit Relational Assessment Procedure (Barnes-Holmes et al., 2006) in measuring sexual orientation among male participants with exclusive sexual interests. The latter task is similar to the IAT but assesses an absolute rather than relative strength of relatedness/association between concepts. Rönspies et al. reported a convergence between viewing time and the Implicit Relational Assessment Procedure, both of which correlated with sexual orientation. The CRT task used showed little evidence of construct validity. This was consistent with the findings of Ó Ciardha and Gormley (2013) but not with the bulk of the published research which appears to demonstrate that the CRT can be used as a valid measure of age-appropriate sexual interest or sexual interest in children (Dombert et al., 2015; Gress, Anderson, & Laws, 2011; Mokros et al., 2010; Santtila et al., 2009; Wright & Adams, 1994).

Using a community sample, the current study examined the convergent validity of viewing time (using three different sets of stimuli) alongside three reaction time tasks: a CRT task, a P-MST, and a variant of the IAT. All tasks included both adult and child stimuli. We tested concurrent validity by examining the relationship between all tasks and self-reported sexual interests in adults and some self-report indicators of possible sexual interest in children. No previous study has simultaneously compared as many measures. Importantly, our study did not solely examine latency and self-report-based measures. We also incorporated a psychophysiological measure of sexual interest into the battery of tasks completed by participants.

The processes that facilitate or hinder speedy task performance in reaction time tasks are typically seen as relatively automatic and, therefore, less vulnerable to faking than other approaches. Where faking occurs, there is evidence that it is detectable and may be correctable (Cvencek, Greenwald, Brown, Gray, & Snowden, 2010). However, latency-based measures require input from the participant in the form of button-presses or vocal responding, which presents an opportunity for noise and/or deliberate manipulation to affect results. We, therefore, also included a psychophysiological method of assessing sexual interest that may be more automatic than tasks requiring a response output from participants. Viewing content that is sexually appetitive elicits an instant automatic dilation of an observer’s pupils consistent with their sexual orientation (Attard-Johnson, Bindemann, & Ó Ciardha, 2016; Rieger et al., 2015; Rieger & Savin-Williams, 2012). Importantly, pupil dilation appears highly resilient to deliberate manipulation (Laeng, Sirois, & Gredebäck, 2012).

The use of pupil dilation as a measure of sexual interest has a long history in the psychophysiology literature but has only recently achieved a level of consistency in findings that supports its validity. Using a somewhat rudimentary measurement approach, Hess, Seltzer, and Shlien (1965) found evidence of pupil dilation to orientation-consistent stimuli among a small sample of gay and straight men. Other researchers (e.g., Scott, Wells, & Wood, 1967), however, failed to replicate their general findings. As a result, this method has been relatively under-researched until recently, when Rieger and Savin-Williams (2012) found, using a large sample, that men’s pupils dilated to erotic video stimuli in line with their sexual interests. Rieger et al. (2015) further established the construct validity of pupil dilation as a measure of sexual interest by demonstrating a correspondence between pupillary responses and genital responses, particularly in males. Using non-nude images rather than videos, Attard-Johnson et al. (2016) found that straight men without a self-reported sexual interest in children showed dilation to adult females but not to images of adult males or children. The current study is the first to examine pupil dilation alongside multiple latency-based measures of sexual interest.

The study had two aims. First, we examined the convergent validity of viewing time, reaction time, and pupillometric measures of sexual interest in a non-offender sample. Participants performed a free-viewing task and three viewing time tasks (with different stimuli) whereby pupil size was recorded, as well as a CRT, a P-MST, and an IAT variant. The IAT variant (which we refer to as a factorial IAT) included adult and child stimuli representing the concept categories of *male* and *female*. We hypothesized that all measures would covary with one another, demonstrating convergent validity, and that they would predict self-reported sexual interest, demonstrating concurrent validity. Second, we examined whether these tasks could also predict sexual interest toward children in this sample, further demonstrating concurrent validity specifically in terms of their forensic application. This second aim was more exploratory given the expectation of a low rate of sexual interest in children in a non-offender population. Santtila et al. (2015), for example, reported a one-year incidence of 3.3% for self-reported sexual interest in children among adult men.

## Method

### Participants

One hundred and two men participated in this study in return for a small payment or course credit.^1^ A sample size target of 100 participants was set based on a combination of experience with previous experiments, pragmatic considerations (e.g., available funding), and a priori power analysis. We calculated the required sample size to test an overall multiple linear regression model with ten predictors of sexual interest in adults with a medium and a large expected effect to equal 118 and 57 participants, respectively. Recruitment materials called for participants for a study ‘comparing eye tracking with other computer-based measures of sexual interests.’ Participants were recruited using a nontargeted approach via the university’s online recruitment system, in addition to more purposive sampling through the university’s student LGBT + society social media page to increase numbers of gay and bisexual men in the samples (for a similar approach, see Rieger & Savin-Williams, 2012).

Mean age was 22.2 years (SD = 5.7; range, 18–50 years). All participants had completed secondary education. Ethnicity was not recorded in this study, but the sample was recruited through advertising in a majority White but ethnically diverse university campus. Participants reported the degree to which they were attracted to male and female adults. The majority were exclusively attracted to female adults (59.8%), 13.7% were predominantly attracted to females, 4.9% were attracted equally to male and female adults, 2.0% were predominantly attracted to male adults, and 19.6% were exclusively attracted to male adults. All participants had normal or corrected-to-normal vision.

#### Stimuli

Pictorial stimuli used across measures were from three sources. The *Not Real People (NRP) set* (clothed version; Laws & Gress, 2004) are composite images of males and females previously developed for use in research involving sexual offenders. Images in the NRP set are presented on a neutral background, classified according to their secondary sexual characteristics (Tanner, 1973), and represent all five Tanner stages. A second set of images (*Morph set*; Ó Ciardha, 2010) provided a greater number of adult (Tanner 5) stimuli than were available using the NRP set alone. These images are realistic but do not represent real people, as faces are morphed composites of different faces. This set included control images of big cats. The third set of images (*Beach set*; Attard-Johnson et al., 2016) consisted of stock photographs portraying adult and prepubescent (Tanner 1) males and females in natural beach scenes (5 images in each of these four categories) and a set of control landscapes without any person content (5 scenes). People were portrayed in swim or leisure wear.

#### Apparatus

##### Pupil Dilation

Tasks that included measurement of pupil dilation were displayed using SR-Research ExperimentBuilder software (version 1.1.0) on a 21” color monitor, with a screen resolution of 1024 × 768 pixels. Eye movements were recorded using an SR-Research Eyelink 1000 eye-tracking system. The Eyelink 1000 was running at a 1000-Hz sampling rate, a spatial resolution of < .01° of visual angle, a gaze position accuracy of < .5°, and a pupil size resolution of .1% of diameter. The Eyelink 1000 system works by measuring corneal reflection and dark pupil with a video-based infrared camera. This system computes the number of camera pixels that are occluded by participants’ pupils and records the area of the pupil as an integer that ranges from 400 to 16,000 units. During the recording of pupil size, participants placed their heads on a chinrest approximately 60 cm from the display monitor.

### Measures

#### Pupil Dilation and Viewing Time Tasks

Participants completed four tasks (one free-viewing and three viewing time tasks) while their pupil responses were recorded. Before commencing each task, the system was calibrated using the standard nine-point fixation Eyelink procedure.

##### Free Viewing of Beach Set Stimuli

In this task, participants were presented with the natural beach scenes. A free-viewing paradigm was adopted whereby participants were instructed to view the images as ‘naturally as they normally would’ (for similar approaches, see Attard-Johnson et al., 2016; Hall, Hogue, & Guo, 2011). Each trial began with a fixation dot, which allowed for drift correction, and ensured that participants attended to the center of the display when the trial began. The trial began with a gray screen which was displayed for 1 s, followed by the stimulus displayed for 10 s, followed by another gray screen for 1 s. Each participant viewed all 25 images once with order randomized across participants.

##### Viewing Time of Beach Set Stimuli

The eye movement setup and procedure was identical to the previous task, except for the following. Scenes without person content were excluded from the beach set stimuli. Instead of being instructed to view images ‘naturally as they normally would,’ participants were instructed to rate the sexual appeal of these targets on a seven-point scale, using the number keys on a standard keyboard. Once a response was registered, the next trial was presented.

##### Viewing Time of NRP Set Stimuli

This task employed the same method and procedure as the previous viewing time task, except that the scene stimuli were replaced with images from the NRP picture set. A total of 40 images were used (four males and four females at each Tanner stage).

##### Viewing Time of Morph Set Stimuli

The procedure was identical to the previous viewing time tasks, except that a total of 36 computer-modified morph set stimuli were used (18 adult males and 18 adult females).

#### Choice Reaction Time Task (CRT)

The CRT was presented using SuperLab4 stimulus presentation software and asked participants to identify as quickly as possible the location of a dot that appeared in one of five positions on an image of a person (or big cats in the practice condition). Stimuli for this task, and for all other reaction time tasks, were presented on a Dell E6520 laptop with a 15.6” color monitor and a screen resolution of 1366 × 768 pixels. Responses were via a Cedrus RB-530 response pad. A white dot (approximately 2.3 mm in circumference) was superimposed on each of five positions on the images, yielding five versions of each image, where the white dot was located either in the top right, top left, bottom right, bottom left, or middle of the image. Each stimulus presentation was preceded by a centrally located fixation cross for 500 ms. Stimuli remained on the screen until participants responded. Participants received no feedback on errors, except for the first 20 practice trials.

Stimuli were taken from the NRP set and the morph set. Images were classified by age as pre-/early pubertal (Tanner 1 and 2), late pubertal (Tanner 3 and 4), and adult (Tanner 5), and by sex (female and male) to yield six trial types. Trial types were presented together in small blocks or clusters of ten images. There were four clusters of each trial type, yielding 40 images of each trial type. Trials were randomized in each cluster, and cluster order was randomized with the constraint that one of each trial type cluster had to be presented before seeing a second of any type and so on. Response times and response correctness were recorded.

#### Pictorial Modified Stroop Task (P-MST)

The P-MST asked participants to identify as quickly as possible, by pressing colored buttons on the response pad, the color in which an image was presented, again using SuperLab4. Images from the NRP and the morph sets had been tinted red, green, blue, and yellow. Four colored versions of twelve images each were used to create blocks of child male, child female, adult male, and adult female images, resulting in 48 images in each block. Unlike the CRT, blocks of child stimuli combined images rated as belonging to Tanner stages 1–4. At the beginning of the task, participants completed a block of 16 practice trials featuring pictures of big cats. Feedback was given for incorrect responses for practice trials but not for the experimental trials. Presentation of stimuli was preceded by a centrally located fixation cross for 500 ms. Stimuli remained on the screen until participants responded. A blocked design was adopted, whereby all adult male images were presented together, as were all adult females, and so on. Order of block presentation was randomized. As with the CRT, response times and response correctness were recorded.

#### Factorial Implicit Association Test (f-IAT)

The f-IAT examined the strength of association between *male*/*female* concept categories and *sexual/nonsexual* attribute categories. Stimuli for the concept category were images from the NRP and morph sets. The f-IAT was based on an IAT used by Ó Ciardha and Gormley (2013), but included images of adults and children resulting in an extra “factorial” element. Twelve male and 12 female images were used, half of adults (Tanner 5) and half of children (Tanner 1–3). The f-IAT was administered over seven blocks. Each stimulus was preceded by a fixation cross presented centrally for 500 ms. Participants could not continue to the next trial until they had answered correctly. Stimuli were presented at least twice within each block.

The first f-IAT block presented stimuli, one at a time, from the concept categories (adult and child images) and participants were instructed to identify them using the left and right buttons on the response pad. Correct category headings were presented in the corresponding left and right corner of the screen. In the second block, participants categorized word stimuli, again using the left and right buttons, as belonging to one of the attribute categories. *Sexual* words were “lust,” “lick,” “kiss,” “naked,” “orgasm,” “arouse,” and “attractive,” while *nonsexual* words were “ugly,” “cold,” “dull,” “avoid,” “bland,” “boring,” and “unattractive.” In the third block, the left-hand concept category from block one was paired with the left-hand attribute category from block two (e.g., *male* and *sexual*), and likewise for the right-hand concept and attribute categories (e.g., *female* and *nonsexual*). Only adult images were presented. The fourth block was identical to the third except that only child images were presented.

In the fifth block, the order of the concept pairs was swapped, whereby *female* might now appear on the left and *male* on the right. Both adult and child images were included in this block. In blocks six and seven, participants categorized both images and words by concept or attribute but with this new arrangement of the concept pairs. In block six, images were of adults, and in block seven images were of children.

Participants’ response times to blocks 3/4 and 6/7 were analyzed as differences between them should indicate differences in the relative strength of associations between the concepts of *male* and *female* and the attributes of *sexual* and *nonsexual*. Furthermore, the strength of these associations could be examined to explore the impact of child stimuli. Order was randomized for participants so that some were presented with male sexual associations first, whereas others were presented first with female-sexual pairings. Blocks three and six contained adult stimuli for all participants, with child stimuli in blocks four and seven.

#### Interest in Child Molestation (ICM) Scale and Problematic Sexual Behavior or Fantasy

The scenarios used in this study were identical to the original ICM scale developed by Gannon and O’Connor (2011), with minor changes to question order and wording. The scale presented five scenarios describing sexual abuse with a child (age and gender unspecified). Participants were asked to imagine themselves in the situation described. They were then asked to answer the following questions on a seven-point scale: (1) “In this situation, how sexually aroused would you be?” (anchor points on the scale were “not at all sexually aroused” and “very strongly sexually aroused”); (2) “In this situation, how much would you enjoy getting your way?” (anchors of “would not enjoy it at all” and “would greatly enjoy it”; and (3) “If you found yourself in a similar situation, would you have done the same?” (anchors of “would definitely not have done the same” and “would definitely have done the same”). The total possible score on the ICM scale ranged from 15 to 105, with the minimum score indicating an emphatic rejection of arousal, enjoyment, or propensity across all child molestation conditions.

Additional questions were included to examine prior sexual offending behavior and sexual fantasy involving children among participants. They were asked if they (1) had ever knowingly and deliberately viewed pornographic material containing individuals below the age of consent, (2) had, since the age of 18, found the thought of sex or sexual contact with a person 15 years of age or younger exciting or arousing, (3) had sexual contact with a child under 13 who was more than 5 years younger than them since the age of 16, or (4) had sexual contact with an individual aged 13–15 who was more than 5 years younger since the age of 18. Only the results of question 1 are presented in the current study.

### Procedure

The study was reviewed and approved by the School of Psychology Research Ethics Committee (REF 20122341). Participants were seated in a windowless room with constant artificial lighting. Participants completed tasks involving pupil dilation first and in the following order: free viewing of beach set, viewing time of beach set, viewing time of NRP set, and viewing time of morph set. Participants then completed the reaction time tasks (CRT, P-MST, and IAT). The order of reaction time tasks was counterbalanced. Finally, participants completed a brief questionnaire to assess sexual interests followed by the ICM scale and questions about problematic sexual behavior or fantasy. In a small number of cases, participants did not complete particular tasks or data did not record properly for technical reasons. We report sample sizes for individual analyses to reflect this. The average length of the entire procedure including obtaining consent, questionnaires, completion of indirect tasks, calibration and administration of eye tracking/pupil dilation paradigms, and debriefing was approximately 90 min per participant.

### Data Preparation

#### Reaction Time Tasks

Reaction time data were calculated based on correct responses only and were trimmed of outliers by removing times more extreme than three times the interquartile range beyond the 25th and 75th percentiles within that experimental condition for that individual. All reaction time data were converted to ipsative *z*-scores. Ipsative *z*-scores present an individual’s mean response times for a given condition in terms of numbers of standard deviations (based on their own data) from their overall mean response time. Therefore, if their response times on a P-MST to images of adult females yields an ipsative *z*-score of + 2, they have been responding slower to images of adult females than their overall mean reaction time by a magnitude of two standard deviations.

Calculating ipsative *z-*scores for the f-IAT differs from the more standard *D* algorithm approach, suggested by Greenwald, Nosek, and Banaji (2003). However, this alternative has been used previously with a similar IAT (Ó Ciardha & Gormley, 2013) and has the strength of being directly comparable with the approach taken with the P-MST and CRT tasks. Additionally, the ipsative *z*-score approach allowed the separate examination of the blocks in which adult and child stimuli were presented.

#### Viewing Time Tasks

As with the reaction time data, viewing time task response times were converted to ipsative *z*-scores. No outlier removal was carried out as there were fewer experimental trials in each condition compared with the reaction time tasks, and participants were not instructed to respond rapidly, meaning even conservative outlier removal risks removing trials that are not inconsistently slow in the context of the task instructions.

#### Pupil Dilation

Observers’ pupillary responses to each stimulus category were calculated as a percentage change from observers’ overall pupil mean. For this, pupillary responses were first computed by taking the mean pupil area at each fixation, averaged across the duration of a stimulus display. An overall mean, across all stimuli in all conditions, was then computed from these values for each participant. The percentage difference (i.e., an increase or decrease) in pupil area for each stimulus category (e.g., adult males) from the overall mean was then computed, using the formula: (mean pupil area for category × 100)/overall pupil mean. Accordingly, a score of 100% indicates that the pupillary response to a stimulus category does not differ from the overall mean. Scores above or below this value indicate comparatively larger or smaller pupil sizes (for similar approaches, see Attard-Johnson et al., 2016; Laeng & Falkenberg, 2007).

## Results

### Assessing Sexual Interest in Adults

For each of the measures (reaction times, viewing times, and pupil dilation), an index of preferred^2^ adult sex was calculated by subtracting the mean responses to adult female stimuli from the mean responses to adult male stimuli (see Table 1). Each of these ten indices were positively correlated, with effect sizes ranging from small to large (Cohen, 1988; see Table 2). Sexual interest measured as a five-point continuous variable (exclusively straight, given a value of 1, to exclusively gay, given a value of 5) positively correlated with the index of preferred adult gender for each measure (*r*s .39–77; see Table 2).

**Table 1:** Samples sizes, grand means, standard deviations, and ranges for indices of sexual interest in adults

	*n*	*M* (SD)	Range	
			Min	Max
Reaction time measures				
P-MST	98	− .12 (.63)	− 1.76	1.68
CRT	100	− .17 (.80)	− 3.33	2.57
f-IAT	100	− .32 (.67)	− 1.63	1.31
Viewing time tasks				
NRP	102	− .81 (1.34)	− 3.21	2.86
Morph	100	− .41 (.96)	− 1.88	1.64
Beach	100	− .39 (.93)	− 2.21	1.54
Pupil dilation				
NRP	100	.27 (6.65)	− 15.93	17.85
Morph	100	− 1.68 (5.18)	− 18.14	8.46
Beach (VT)	100	− 2.6 (7.14)	− 18.00	14.77
Beach (free)	100	− 3.51 (6.48)	− 19.13	14.69

P-MST, pictorial modified Stroop task; f-IAT, factorial Implicit Association Test; NRP, not real people image set (Laws & Gress, 2004); VT, viewing time; reaction and viewing time values represent the mean difference in ipsative *z*-scores between responses to adult male versus adult female stimuli; pupil dilation values represent the mean difference between pupillary responses to adult male versus adult female stimuli as a percentage of individual mean pupil size. For all measures, negative means suggest greater sexual interest in female adults

**Table 2:** Correlation coefficients between indices of preferred adult sex and self-reported adult sexual interests

		1	2	3	4	5	6	7	8	9	10
Reaction time measures											
1. P-MST											
2. CRT	*r*	.62									
	*n*	98									
	BCa 95% CI	[.44, .74]									
3. f-IAT	*r*	.49	.52								
	*n*	98	100								
	BCa 95% CI	[.33, .61]	[.40, .64]								
Viewing time tasks											
4. NRP	*r*	.49	.43	.53							
	*n*	98	100	100							
	BCa 95% CI	[.38, .59]	[.29, .56]	[.38, .66]							
5. Morph	*r*	.55	.40	.50	.83						
	*n*	96	98	98	100						
	BCa 95% CI	[.41, .66]	[.26, .53]	[.31, .65]	[.76, .89]						
6. Beach	*r*	.43	.37	.40	.67	.72					
	*n*	96	98	98	100	100					
	BCa 95% CI	[.20, .61]	[.11, .58]	[.21, .57]	[.55, .77]	[.61, .81]					
Pupil dilation											
7. NRP	*r*	.42	.37	.40	.64	.65	.48				
	*n*	96	98	98	100	100	100				
	BCa 95% CI	[.28, .53]	[.22, .53]	[.23, .54]	[.49, .75]	[.52, .76]	[.32, .62]				
8. Morph	*r*	.44	.46	.53	.68	.68	.55	.66			
	*n*	96	98	98	100	100	100	100			
	BCa 95% CI	[.31, .57]	[.33, .60]	[.39, .65]	[.57, .77]	[.57, .77]	[.41, .68]	[.50, .77]			
9. Beach (VT)	*r*	.14	.29	.22	.39	.43	.45	.32	.49		
	*n*	96	98	98	100	100	100	100	100		
	BCa 95% CI	[− .04, .32]	[.04, .52]	[.03, .40]	[.23, .53]	[.28, .57]	[.30, .59]	[.13, .49]	[.34, .62]		
10. Beach (free)	*r*	.15	.20	.23	.30	.39	.31	.29	.39	.38	
	*n*	96	98	98	100	100	100	100	100	100	
	BCa 95% CI	[− .002, .31]	[.02, .39]	[.05, .40]	[.11, .48]	[.24, .53]	[.13, .46]	[.11, .46]	[.23, .54]	[.17, .54]	
Sexual orientation	*r*	.59	.46	.65	.72	.77	.58	.58	.69	.39	.44
	*n*	98	100	100	102	100	100	100	100	100	100
	BCa 95% CI	[.46, .69]	[.32, .58]	[.52, .75]	[.58, .83]	[.67, .85]	[.44, .69]	[.44, .70]	[.59, .77]	[.24, .52]	[.29, .59]

P-MST, pictorial modified Stroop task; f-IAT, factorial Implicit Association Test; NRP, not real people image set (Laws & Gress, 2004); VT, viewing time; BCa 95% CI, 95% confidence intervals based on 5000 resamples using the bias-corrected and accelerated (BCa) bootstrap method

A multiple linear regression model incorporating all indices of preferred adult gender explained 75% of the variance in sexual interest (see Table 3). This amount of variance explained equates to a large effect size. However, it also demonstrates that there was still some error in the ability of the indices of preferred gender to predict self-reported sexual interest. We explored whether a source of error might stem from cases where individuals reported non-exclusive sexual interests, by examining whether exclusivity moderated the relationship between predicted values in the regression model and observed sexual interest (all variables standardized), using the PROCESS macro for SPSS (model 1; Hayes, 2013). The analysis (*n* = 96) showed that the relationship between predicted and observed values was weaker for those with non-exclusive sexual interests, *β* = .32; SE = .18; BCa 95% CI [− .04, .67], compared with exclusive sexual interests, *β* = .91; SE = .05; BCa 95% CI [.80, 1.01].

**Table 3:** Summary of multiple linear regression analysis examining whether indices of preferred adult sex predict sexual orientation (on a five-point scale)

	*B*	SE *B*	*β*	BCa 95% CI
Reaction time measures				
P-MST	.59	.22	.23	[.15, 1.03]
CRT	− .22	.16	− .10	[− .54, .09]
f-IAT	.55	.14	.23	[.25, .83]
Viewing time tasks				
NRP	.22	.16	.19	[− .11, .56]
Morph	.44	.22	.27	[.001, .87]
Beach	− .11	.13	− .07	[− .36, .12]
Pupil dilation				
NRP	.01	.02	.02	[− .04, .05]
Morph	.05	.03	.18	[− .01, .12]
Beach (VT)	− .002	.02	− .01	[− .04, .03]
Beach (free)	.03	.02	.14	[.002, .07]
*R* ^2^				.75
*F*				25.82
*n*				96

P-MST, pictorial modified Stroop task; f-IAT, factorial Implicit Association Test; NRP, not real people image set (Laws & Gress, 2004); VT, viewing time; *B*, unstandardized regression coefficient; *β*, standardized regression coefficient; BCa 95% CI, 95% confidence intervals of *B* based on 5000 resamples using the bias-corrected and accelerated (BCa) bootstrap method

### Assessing Sexual Interest in Children

The mean interest in child molestation (ICM) score for this sample was 20.6 (SD = 9.3; range 15–59), with just over half the sample (51%) emphatically rejecting an interest in child sexual abuse (i.e., scoring 15). Additionally, 11.8% (*n* = 13) admitted knowingly and deliberately viewing pornographic material containing individuals below the age of consent. Despite non-minimal responding in the ICM and admission of the use of pornographic material containing children by some participants, it is unlikely that the sample would contain enough individuals with a clear sexual interest in children to robustly examine whether the tasks were valid measures of preferred age as well as preferred sex. However, it was possible to carry out preliminary investigations to examine whether participants who appeared to demonstrate sexual interest in younger individuals—through increased attention, stronger sex associations, or greater pupil dilation—also self-reported greater interest in child molestation or were more likely to report having previously used pornographic material containing children.

We coded our predictor variables into binary values indicating whether individuals’ strongest average response was to adult stimuli (Tanner 5) or to younger stimuli.^3^ Table 4 shows the percentage of participants with a stronger response to younger stimuli across all tasks alongside rank-biserial correlations with the ICM. The binary predictors of adult or child interest were entered into a multiple linear regression predicting ICM scores (see Table 5). Overall, the model accounted for 21% of the variance in ICM scores. This reflects a moderate effect size.

**Table 4:** Percentage of participants with a stronger average indirect task response to child stimuli and rank-biserial correlation between having a stronger response to children and ICM scores

	*n*	Percentage of strongest response to child stimuli (%)	Correlation with ICM	
			*r*_rb_	BCa 95% CI
Reaction time measures				
P-MST	98	29.6	.07	[− .14, .28]
CRT	100	56.0	.08	[− .12, .26]
f-IAT	100	44.0	.04	[− .16, .24]
Viewing time tasks				
NRP	102	40.2	− .02	[− .21, .18]
Beach	100	8.0	.25	[.003, .44]
Pupil dilation				
NRP	100	83.0	.32	[.16, .45]
Beach (VT)	100	13.0	.10	[− .11, .30]
Beach (free)	100	14.0	.05	[− .15, .26]

P-MST, pictorial modified Stroop task; f-IAT, factorial Implicit Association Test; NRP, not real people image set (Laws & Gress, 2004); VT, viewing time; *r*_rb_, rank-biserial correlation coefficient; BCa 95% CI, 95% confidence intervals based on 5000 resamples using the bias-corrected and accelerated (BCa) bootstrap method

**Table 5:** Summary of multiple linear regression analysis examining whether indices of preferred age predict ICM scores

	*B*	SE *B*	β	BCa 95% CI
Reaction time measures				
P-MST	1.54	2.40	.08	[− 3.06, 6.74]
CRT	1.79	1.83	.10	[− 1.71, 5.45]
f-IAT	1.20	1.87	.06	[− 2.54, 4.88]
Viewing time tasks				
NRP	− .97	1.86	− .05	[− 4.83, 2.67]
Beach	10.32	5.46	.31	[.13, 22.00]
Pupil dilation				
NRP	5.80	1.37	.23	[3.53, 8.36]
Beach (VT)	2.28	2.66	.08	[− 2.10, 6.90]
Beach (free)	− 1.02	1.99	− .04	[− 5.23, 3.09]
*R* ^2^				.21
*F*				2.94
*n*				96

P-MST, pictorial modified Stroop task; f-IAT, factorial Implicit Association Test; NRP, not real people image set (Laws & Gress, 2004); VT, viewing time; *B*, unstandardized regression coefficient; β, standardized regression coefficient; BCa 95% CI, 95% confidence intervals of *B* based on 5000 resamples using the bias-corrected and accelerated (BCa) bootstrap method

In our sample, there were too few participants who admitted having viewed pornographic material containing children to examine multiple predictors of that outcome in a logistic regression. Therefore, we examined the relationship between indirect measures and use of pornographic material containing children using a *t* test. The dependent variable in this case was the total number of tasks showing a stronger response to child stimuli compared to adults. Across the eight paradigms, individuals who admitted use of pornographic material containing children had a greater number of strongest responses to child stimuli (*M* = 3.8, SD = 1.5) compared with individuals who reported no use (*M* = 2.8, SD = 1.2). This difference, − .99, BCa 95% CI [− 1.91, − .06], represented a large effect size, albeit with a wide confidence interval; *t*(94) = − 2.57, *d* = .80 (effect size calculated using supplementary materials from Lakens, 2013).

## Discussion

In our sample, indices of sex preference to adult stimuli across tasks were consistently related to one another and to self-reported sexual interest toward adults, consistent with our hypotheses. All correlations between indices were positive though effect sizes varied from small to large. Correlations between indices of sex preference and self-reported sexual interest toward adults were again positive, with effect sizes ranging from moderate to large. These relationships demonstrated evidence of the convergent and concurrent validity of latency-based tasks and of pupil dilation.

Combined indices of sex preference to adult stimuli predicted 75% of the variance in self-reported sexual interest in adults. While some predictors did not uniquely contribute to the model’s overall ability to predict self-reported sexual interest, those that did spanned reaction time, viewing time, and pupil dilation approaches. In a post hoc analysis, we found that exclusivity of sexual interest moderated the strength of the relationship between the predicted values and the observed value in our regression model above. This finding appears consistent with the finding by Rieger et al. (2015) of weaker correspondence between indices of arousal in bisexual men compared to gay or straight men.

Our examination of measures as predictors of sexual interest in children was exploratory given the expectation of low numbers of participants with sexual interest in children. To determine whether indirect task performance suggested a sexual interest in children, we created post hoc dichotomous variables indicating whether the strongest response on any given indirect measure was to child or adult stimuli. Table 5 shows the percentage of individuals showing a strongest response to child stimuli across indirect measures, and how measures correlate with ICM scores. It is noteworthy that the percentage of participants showing a stronger response to children varied considerably depending on the task. This is a point we return to later. Indirect tasks performance, dichotomized in this way explained 21% of the variance in ICM scores, corresponding to a moderate effect size. Knowingly and deliberately viewing pornographic material containing children was related to having stronger response to children in a greater number of tasks. Taken together, and interpreted in the context of a low base rate of pedophilia and hebephilia in community samples, these results are very preliminary indicators of concurrent validity of the tasks as measures of sexual interest in children.

Our findings offer evidence that indirect measures of sexual interest operate predictably and in a manner that is consistent with one another, particularly with adult stimuli. This was true of the cognitive and psychophysiological paradigms examined in the current study, at least in the conditions experienced by our participants. Different processes relating to sexual attention or arousal are likely to underpin these effects depending on the task (Ó Ciardha, 2011). Pupil dilation, for example, is likely to reflect increased activity in the sympathetic nervous system (Bradley, Miccoli, Escrig, & Lang, 2008). IAT, on the other hand, measures the strength of association between simple concepts held in memory and therefore possibly reflects schematic relationships in ‘implicit sexual memory’ (for a discussion of explicit and implicit sexual memory, see Spiering & Everaerd, 2007).

The CRT and P-MST appear to be measures of selective attention. However, both these tasks in the current study did not randomly present individual trials but rather combined stimuli of the same trial type into blocks or clusters of stimuli. This follows recommendations for maximizing modified Stroop effects with appetitive stimuli (specifically addiction Stroop tasks, see Cox, Fadardi, & Pothos, 2006). With a blocked design, it is not possible to examine whether differences in latencies are due to an instantaneous capturing of attention or because of carryover effects once a given stimulus has been erased. As a result, while the current study provided evidence of criterion validity across this battery of tasks by demonstrating their convergence, research examining their discriminant validity is needed to better understand the processes underpinning individual tasks.

In the current study, tasks containing child images did not all have identical groupings of stimuli by stage of sexual development. This was a consequence of selecting tasks that had previously been piloted or otherwise tested within our laboratory as part of existing research streams. It is noteworthy that the measure suggesting the highest rates of interest in children among our sample (83%) was the pupil dilation method using NRP stimuli. The tasks using NRP stimuli were the only tasks which generated response data for each Tanner stage individually. Inspection of the raw data showed that the percentage was driven in large part by the number of participants (31%) showing the greatest average pupil dilation to Tanner 4 stimuli.

Ebsworth and Lalumière (2012) described Tanner 4 as representing “individuals who are on the cusp of reaching full sexual maturity” (p. 164). As a result, a sexual interest in Tanner 4 images may not be a particularly useful index of forensically relevant sexual interest, especially given the low average age of our participants. Despite this limitation, however, the pupil dilation method using NRP stimuli was a predictor of ICM scores, with inspection of descriptive data indicating that participants showing the strongest pupil dilation to Tanner 5 stimuli also had the lowest ICM scores. Future studies using indirect measures should be designed, where possible, to measure the relative strength of response to stimuli across the full range of sexual maturity.

The most robust findings of our study were those establishing convergent and concurrent validity across tasks measuring sexual interest using adult stimuli, particularly where participants reported exclusive sexual interests in one sex or another. The more exploratory findings examining sexual interest in children will require corroboration with future studies containing greater number of pedophilic and hebephilic participants. However, establishing criterion validity of sexual interest tasks for forensic use based on age-appropriate sexual interest using non-offending samples is an important step. Seto (2017), for example, outlined a multidimensional view of sexual orientation, defining sexual orientation as a “stable tendency to preferentially orient—in terms of attention, interest, attraction, and genital arousal—to particular classes of sexual stimuli” (p. 3).

Seto (2017) argued that while sex is the usual dimension along which sexual orientation is discussed, age, or more specifically physical and sexual maturity, represents a dimension on which people may hold a stable orientation. He referred to variations in individual orientation on this age dimension as *chronophilias*, which include age preferences to prepubescent children (pedophilia), pubescent children (hebephilia), young adults (teleiophilia), etc. Within this multidimensional model, sexual interests may differ across individuals in terms of what is attractive, but may not differ in how that attraction impacts attention and arousal (see also Ó Ciardha, 2011). Therefore, results when measuring teleiophilic sexual interest among men (e.g., task validity) should generalize to the testing of male sexual interest toward other age groups. If they do not, there are implications for the conceptualization of pedophilia, for example, as dimensionally related to a sexual interest in adults.

Data were collected in the current study under non-adversarial conditions. As a result, we cannot be certain which measures are most resilient to faking or whether there are certain conditions under which some or all measures do not correspond to an individual’s sexual interest (i.e., discriminant validity). However, we are least enthusiastic about the potential utility of the viewing time approach, as it is the most transparent methodology. Schmidt et al. (2017) state similar concerns and lament the lack of fakeability studies. While knowledge of reaction time tasks such as the P-MST, CRT, and f-IAT would theoretically also allow people to manipulate their responses, the fact that rapid responses are required makes it less likely that participants could produce a coherent but fake pattern of responding. Given that pupil dilation is an automatic response to activity in the sympathetic nervous system (Bradley et al., 2008), it is likely to be the task that is least susceptible to deliberate manipulation (Laeng et al., 2012). Future research should address issues of fakeability, discriminant validity, and of course include participants with an admitted sexual interest in children. However, taken together our results are the first to demonstrate strong evidence of the convergent and concurrent validity of viewing time, reaction time, and pupillometric approaches to the measurement of sexual interest.

## Footnotes

1 Attard-Johnson et al. (2017) presented a detailed examination of the pupil dilation data of 100 participants from this data set.

2 For reasons of parsimony, we used the word *preferred* with reference to gender or age in describing whether response time or pupil dilation tasks indicated greater sexual interest to stimuli of a particular sex or age across individuals. We used the word to indicate the relative strength of associations, pupil dilations, etc., to different stimuli, not to imply voluntary choice in sexual orientation (see Committee on Lesbian and Gay Concerns, American Psychological Association, 1991).

3 Some previous studies combine Tanner 4 and 5 images as representing mature (e.g., Ebsworth & Lalumière, 2012) or have omitted Tanner 4 images entirely (e.g., Banse, Schmidt, & Clarbour, 2010). We were unable to isolate Tanner 4 images across all paradigms presented. We therefore examined only whether individuals responded most strongly to Tanner 5 versus all other Tanner stages. We examine implications in the Discussion section.
